# Effects of Crumb Rubber Content and Preparation Temperature on the Asphalt Performance and Fume Emissions of Deodorized Rubberized Asphalt

**DOI:** 10.3390/ma19071460

**Published:** 2026-04-05

**Authors:** Wenxiu Wu, Xiangzheng Fang, Yonglin Hu, Huiyi Jin, Yinyan Li, Yifei Sun, Wanyu Wu, Chao Li, Yingjun Jiang

**Affiliations:** 1 College of Civil and Transportation Engineering, Hohai University, Nanjing 210024, China; wenxiuwu123@163.com; 2 Jinhua Highway and Transportation Management Center, Jinhua 321000, China; liyinyan12@163.com (Y.L.); 18256510176@163.com (Y.S.); 3 Jindong District Highway and Transportation Management Center, Jinhua 321000, China; 17868800293@163.com (Y.H.); huiyijin2026@163.com (H.J.); 4 School of Highway, Chang’an University, Xi’an 710064, China; wanyuwu21@163.com (W.W.); chaoli2026@163.com (C.L.)

**Keywords:** deodorized rubberized asphalt, crumb rubber content, preparation temperature, asphalt performance, fume emissions

## Abstract

While rubberized asphalt with a crumb rubber content of 20% to 40% can improve asphalt performance, it also faces prominent issues such as increased construction viscosity and intensified fume emissions. Currently, systematic studies on high-content deodorized rubberized asphalt across different preparation temperatures remain insufficient, particularly regarding the synergistic optimization of performance enhancement and fume emission control, including gaseous pollutants and particulate matter. To address this, deodorized crumb rubber (G-CR), a surface-treated crumb rubber produced by coating with a deodorizing agent, was introduced in this study and blended with base asphalt to prepare deodorized rubberized asphalt (G-CRA). Through laboratory binder tests, the coupled effects of crumb rubber content and preparation temperature (170–200 °C) on the conventional properties, rheological characteristics, and fume emissions of G-CRA were systematically analyzed. The results show that at 30% crumb rubber content and 190 °C preparation temperature, the asphalt binder achieves an optimal balance among penetration, ductility, and softening point, along with significantly improved high-temperature stability and aging resistance. Compared to conventional crumb rubber asphalt (CRA, without deodorant treatment), G-CRA achieves a significant reduction in fume emissions, with SO_2_ reduction reaching up to 81%. This study demonstrates that deodorized crumb rubber can effectively synergize performance enhancement and gaseous emission control under high-content conditions, providing laboratory-level data support for the development of environmentally friendly rubberized asphalt.

## 1. Introduction

The extensive accumulation of waste tires represents a critical environmental challenge on a global scale. The transformation of waste tires into crumb rubber (CR) for use in asphalt modification serves a dual purpose: facilitating waste recycling and enhancing asphalt performance. This approach has garnered considerable research interest [1,2,3]. Prior investigations have consistently demonstrated that both the proportion and particle size of crumb rubber (CR) substantially influence the high-temperature stability, fatigue resistance, and ductility of asphalt and also contribute to the mitigation of traffic noise. For example, Martina et al. [4] found that rubber-modified asphalt reduced traffic noise by up to 3.3 dB compared to conventional asphalt, with the benefit remaining stable over time. The sustainable objective pursued by this technical pathway aligns with the rationale behind utilizing industrial by-products such as fly ash and magnesium slag for cementitious materials [5,6]. Beyond waste valorization, recent life cycle assessment (LCA) studies have systematically evaluated the environmental performance of rubberized asphalt pavements across key life cycle stages. Wang et al. [7] demonstrated that incorporating waste engine oil residue and crumb rubber into recycled asphalt during the material production stage reduced global warming potential by 79.6% and non-biotic resource consumption by 90.8%. Gamboa et al. [8] noted that due to improved workability, rubberized asphalt reduces raw material usage and mix production energy during the construction phase. In addition, the same study found that rubberized asphalt pavements achieve a 2.8 dB reduction in traffic noise during the service stage, contributing to a 67% lower impact on human health compared to conventional asphalt. Farina et al. [9] emphasized that in terms of maintenance, the enhanced durability of rubberized mixtures significantly reduces the need for rehabilitation interventions, leading to a carbon footprint 69–84% lower than that of unmodified mixtures when long-term performance is considered. Collectively, these findings highlight the significant environmental and economic benefits of rubberized asphalt throughout its life cycle, establishing a solid foundation for its broader adoption in sustainable road infrastructure. However, compared to these systems, rubberized asphalt remains significantly constrained in practical applications by key bottlenecks such as construction workability and storage stability.

Simultaneously, the emission of fumes during the production and construction stages of crumb rubber asphalt (CRA) has attracted growing academic interest. Elevated processing temperatures markedly intensify pollutant emissions, and the incorporation of crumb rubber (CR) leads to increased release of volatile organic compounds (VOCs), sulfides, and carbon monoxide (CO) compared to conventional base asphalt [10,11,12]. As a result, emission control has emerged as a significant obstacle to the widespread adoption of CRA with a rubber content exceeding 20% [13]. To address this issue, Borinelli et al. [14,15] demonstrated that emission-reduction agents can effectively suppress VOC emissions from CRA. However, their subsequent research indicated that the wet addition of crumb rubber and inhibitors may influence binder rheology. Therefore, a careful balance between emission mitigation and material performance is necessary. In a study by Wang et al. [16], VOC emission reduction techniques in asphalt were reviewed, with emission reduction agents categorized into organic polymers, inorganic materials, and composites, and future research directions identified as laboratory-field correlation and the development of eco-friendly agents. Cao et al. [17] further revealed that various inhibitors substantially modify VOC emission profiles, thereby mitigating environmental odor and photochemical smog pollution and reducing the risk of carcinogenicity and neurological damage to human health. Additionally, Lv and Wu et al. [18,19] established that UiO-66, a metal–organic framework (MOF) with high specific surface area and controllable structure, effectively reduces VOC emissions via selective adsorption mechanisms. Moreover, Zhang et al. [20] found that coating crumb rubber with waste cooking oil enhanced dispersion, improved storage stability, and decreased sulfide emissions. Despite these advancements, current approaches predominantly focus on singular additives or post-treatment solutions, yet seldom explore the interplay between deodorization and high rubber content, the coordinated optimization of key preparation parameters (e.g., content and temperature), or the mechanistic links between emission reduction and material properties. Consequently, there remains a significant lack of systematic evaluation of high-content deodorized CRA, especially regarding its integrated performance under varied processing conditions.

Extensive investigations have been undertaken to assess the performance attributes of systems containing high proportions of rubber. Wang et al. Wang et al. [21] demonstrated that increasing crumb rubber content substantially elevates the softening point and enhances rutting resistance in asphalt binders. Additionally, Khalili et al. [22] conducted a systematic characterization of CR-modified binders, confirming enhanced viscoelastic properties at elevated temperatures. Yang et al. [23] and Ouyang et al. [24], employing atomic force microscopy (AFM) techniques, revealed alterations in micromechanical properties, phase distribution, and adhesion following aging. Ji et al. [25] emphasized that aging significantly modifies phase structure and modulus in binders with high rubber content, whereas Lyu et al. [26] underscored the necessity of balancing performance improvements with environmental considerations. Kantatham et al. [27] demonstrated that the incorporation of rubber latex and bottom ash significantly enhances both the compressive and flexural strength of cement concrete pavements. Concurrently, Bashir et al. [28] highlighted that rubberized asphalt, as a green building material, is widely utilized in pavement construction in developing countries and contributes notably to the improvement of asphalt performance. In addition, recent studies have further investigated the effects of crumb rubber content and temperature on the rheological properties of rubberized asphalt. Kim et al. [29] evaluated binders with rubber contents of 10%, 15%, and 20%, explicitly discussing their temperature dependence. Li et al. [30] systematically compared the physical and rheological properties of asphalts with rubber contents of 10%, 15%, 20%, and 25%. Al-Khateeb et al. [31] reported systematic changes in binder properties as rubber content increased from 0% to 20%. Badri et al. [32] employed response surface methodology to analyze the effects of rubber content ranging from 5% to 15% and its interaction with high-temperature conditions. Although these studies have provided valuable insights into the performance of rubberized asphalt, systematic evaluations of high-content (20–40%) deodorized rubberized asphalt under varying preparation temperatures remain limited. In particular, the synergistic optimization of performance enhancement and toxic fume emission control, as well as the interplay among crumb rubber content, preparation temperature, and deodorization treatment, has yet to be comprehensively explored.

In summary, existing studies substantiate the advantages of CRA in enhancing asphalt performance while simultaneously identifying challenges related to emissions and constructability. However, systematic investigations focusing on high-content deodorized CRA across varying preparation temperatures remain scarce.

Hence, this study aims to examine the influence of crumb rubber content and preparation temperature on conventional properties, Brookfield viscosity, short-term aging behavior, rheological characteristics, and fume emissions in order to elucidate the synergistic mechanisms between asphalt performance and environmental factors in high-content deodorized CRA and thus support accurate future pavement performance evaluation. The findings are expected to provide theoretical insights supporting sustainable pavement engineering practices. China’s Air Pollution Prevention and Control Action Plan and the 14th Five-Year Plan prioritize the control of atmospheric pollutants, including VOCs [33]. However, specific emission limits for rubberized asphalt pavement construction remain undefined. Therefore, this study provides a practical reference for the formulation of future environmental standards in this field.

## 2. Materials and Methods

### 2.1. Materials

(1) Base asphalt

The base asphalt used was 70# petroleum asphalt produced in Maoming, Guangdong. The key technical properties of the binder are summarized in Table 1, where the classification basis for the asphalt grade and the specification requirements for the test results are both defined by the Chinese standard [34].

(2) Crumb rubber

The experimental study employed deodorized crumb rubber (G-CR) produced by Shenzhen Luhaiwei Material Technology Co., Ltd. (Shenzhen, China), with conventional crumb rubber (CR) from Hebei Lingshou Shengfei Mineral Products Processing Plant (Shijiazhuang, China) serving as the control group. Both types of crumb rubber were uniformly selected with a 60-mesh specification.

The deodorized crumb rubber was prepared by coating conventional crumb rubber with a proprietary deodorizing agent. According to the manufacturer, the deodorizing agent is a composite formulation primarily composed of surfactants and metal oxides, which is applied via a spraying and drying process.

The surface of conventional CR exhibits a typically rough and irregular morphology resulting from mechanical grinding, whereas G-CR, after being coated with the deodorizing agent, shows a rough, porous, and wrinkled surface structure. The main technical indicators are listed in Table 2 and Table 3.

(3) Additive

Aromatic oil was used as an additive. According to the manufacturer, it is rich in aromatic hydrocarbons and exhibits good compatibility and solubility with rubber molecular chains, which facilitates the penetration and swelling of rubber particles during mixing. This is believed to promote the dispersion of rubber particles and improve interfacial compatibility with the asphalt matrix. The recommended dosage provided by the manufacturer is 5% by mass of the base asphalt. The main properties of the additive are presented in Table 4.

### 2.2. Methods

#### 2.2.1. Conventional Performance Tests

Fundamental properties of the binder, including penetration, ductility, and softening point, as well as construction viscosity measured at 135 °C and 180 °C using Brookfield viscometry, were systematically examined. Short-term aging resistance was evaluated using the Thin Film Oven Test (TFOT), which was selected over the Rolling Thin Film Oven Test (RTFOT) due to the high viscosity of the rubberized binders at high crumb rubber contents, which precludes the use of the rolling film procedure. High-temperature rheological behavior was assessed using a Dynamic Shear Rheometer (DSR). All tests were conducted in accordance with the Chinese standard [35].

#### 2.2.2. Rheological Tests

A Dynamic Shear Rheometer (DSR) was utilized to assess the rheological behavior of the binders. Temperature sweep tests were performed on unaged binders using a 25 mm parallel plate geometry with a 1 mm gap. Measurements were conducted at an angular frequency of 10 rad/s within the linear viscoelastic strain amplitude (12%) at temperatures of 58, 64, 70, 76, 82, and 88 °C. The magnitude of the complex shear modulus |G*|, phase angle δ, and rutting parameter |G*|/sinδ were determined accordingly.

#### 2.2.3. Scanning Electron Microscopy (SEM) Analysis

The surface morphology of the rubberized asphalt samples was observed using a JSM IT800 scanning electron microscope (JEOL Ltd., Tokyo, Japan). Prior to observation, the samples were prepared according to standard test methods, mounted onto aluminum stubs using conductive carbon tape, and sputter-coated with a gold layer of approximately 10 nm under vacuum to enhance conductivity and prevent charging effects. SEM imaging was performed at an accelerating voltage of 20 kV with a working distance of 10–15 mm.

#### 2.2.4. Preparation Procedure

Rubberized asphalt was prepared using a three-step process of pre-mixing, high-shear shearing, and development [36]. First, crumb rubber and base asphalt were blended at the specified temperature using an electric stirrer at 600 r/min for 0.5 h to ensure initial dispersion of the rubber particles. The pre-mixed blend was then sheared with a high-speed shear mixer at 5000 r/min for 1 h to achieve thorough interaction between the modifier and the binder. Finally, the sheared sample was continuously stirred at 600 r/min for 2 h using an electric stirrer to complete the development stage. The detailed preparation parameters are summarized in Table 5.

#### 2.2.5. Fume Emission Measurement

**Figure 1:**
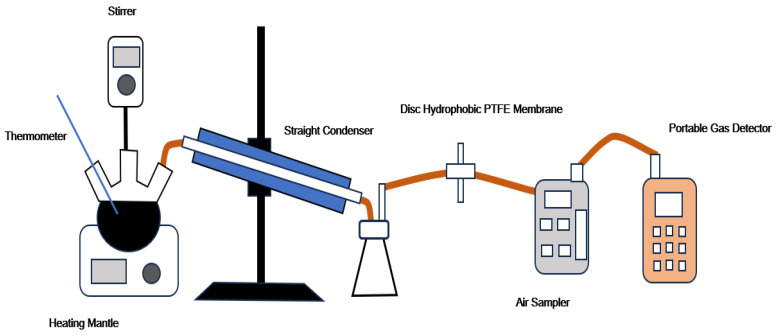
Schematic of the asphalt fume collection device.

The fume emission test setup consisted of a heating and fume generation unit and a collection unit, as illustrated in Figure 1.

The heating unit included a thermostatically controlled heating mantle, a three-neck flask, a mechanical stirrer, and a thermometer. The collection unit was composed of a hydrophobic polytetrafluoroethylene (PTFE) membrane filter (diameter: 50 mm; pore size: 0.22 μm), a conical flask, an atmospheric sampler, and a portable multi-gas detector. A cooling system comprising a condenser and a conical flask was integrated to maintain the detection temperature below 70 °C.

During each test, asphalt fumes were drawn by the sampler and sequentially passed through the PTFE membrane filter and the gas detector. The concentrations of hydrogen sulfide (H_2_S), nitric oxide (NO), carbon monoxide (CO), volatile organic compounds (VOCs), sulfur dioxide (SO_2_), carbon dioxide (CO_2_), and particulate matter (PM) were continuously monitored. Measurements were recorded at the third minute of heating at 180 °C. All tests were conducted in triplicate to ensure reproducibility, and the average values were reported.

Prior to each measurement series, the gas detectors were calibrated using standard reference gases, and a blank test was performed under identical conditions without an asphalt sample to correct for background interference. The manufacturer-specified uncertainties for the gas detectors were as follows: VOCs: ±2% of reading, H_2_S: ±3%, NO: ±3%, SO_2_: ±3%, CO: ±2%, CO_2_: ±2%, and PM: ±5% of reading. Gas flow rates were maintained below 1 L/min, with detection ranges as follows: VOCs: 0–300 ppm, H_2_S: 0–100 ppm, NO: 0–100 ppm, SO_2_: 0–100 ppm, CO: 0–1000 ppm, CO_2_: 0–10,000 ppm, and PM: 0–10 g.

The reduction rate of pollutant emissions was calculated using the following equation:

(1) $$R=\left(\frac{E_{CRA}-E_{G-CRA}}{E_{CRA}}\right)\times 100\%$$

where *R* is the proportion of pollutant emission reduction, used to evaluate the mitigation effect of the G-CRA compared with the CRA, *E_CRA_* is the pollutant emission of conventional crumb rubber asphalt, and *E_G__−CRA_* is the pollutant emission of odorless crumb rubber asphalt.

The particulate matter content was calculated from the weight difference in the PTFE membrane before and after testing.

## 3. Effect of Crumb Rubber Content and Preparation Temperature on the Properties of Rubberized Asphalt

The standard characteristics of rubberized asphalt at varying preparation temperatures (ranging from 170 to 200 °C) and crumb rubber concentrations (20 to 40%) are detailed in Table 6 and Table 7.

### 3.1. Conventional Properties

(1) Penetration

**Figure 2:**
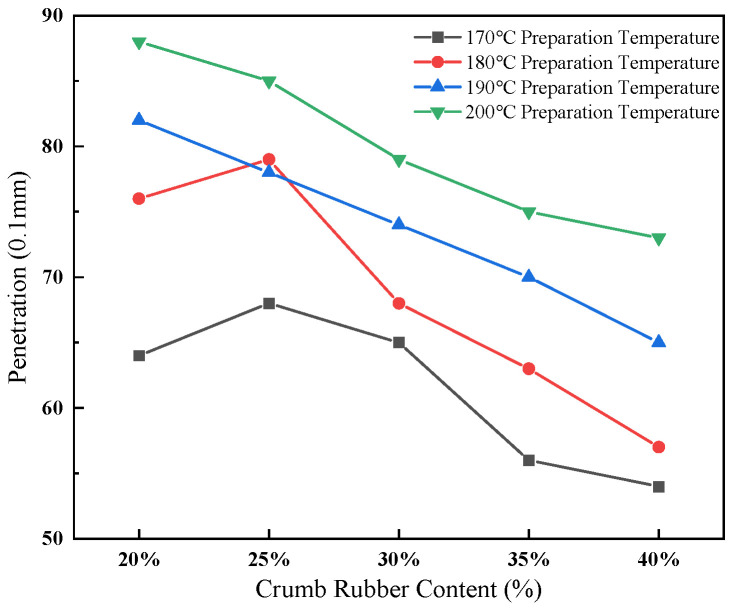
Effect of crumb rubber content and preparation temperature on penetration of rubberized asphalt.

The effect of crumb rubber content and preparation temperature on penetration is shown in Figure 2.

Figure 2 depicts the effects of crumb rubber content and preparation temperature on penetration values. The data indicate a significant increase in penetration with rising temperature; for instance, at 20% rubber content, penetration increased from 64 at 170 °C to 88 at 200 °C. In contrast, penetration values decreased as the rubber content increased; specifically, at 180 °C, penetration declined from 76 at 20% rubber content to 57 at 40%. These findings suggest that elevated temperatures tend to soften the asphalt matrix, while higher rubber content contributes to increased binder stiffness. Overall, maintaining a moderate preparation temperature range of 180–190 °C in conjunction with a rubber content of 25–30% appears to achieve an optimal balance between hardness and workability.

(2) Ductility

**Figure 3:**
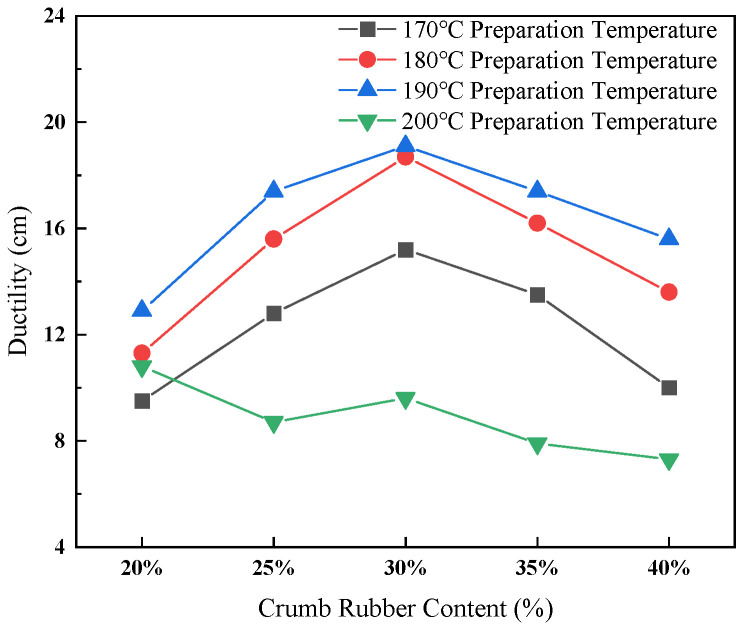
Effect of crumb rubber content and preparation temperature on ductility of rubberized asphalt.

The effect of crumb rubber content and preparation temperature on ductility is presented in Figure 3.

As illustrated in Figure 3, ductility initially increased and subsequently decreased with variations in both temperature and rubber content. The peak ductility value of 19.1 cm was observed at a rubber content of 30% and a temperature of 190 °C, indicating adequate swelling of the crumb rubber and the formation of a robust asphalt–rubber network, which enhanced low-temperature flexibility. Conversely, a marked decline in ductility was noted at 200 °C, suggesting significant degradation of the rubber at elevated temperatures. Huang et al. [37] investigated rubber asphalt with rubber contents of 20%, 30%, 40%, and 50% and reported corresponding ductility values of 5.4 cm, 17.7 cm, 16.4 cm, and 10 cm, respectively. It can be seen that ductility first increased and then decreased with increasing rubber content, reaching a maximum near 30%, which is consistent with the trend observed in this study.

(3) Softening point

**Figure 4:**
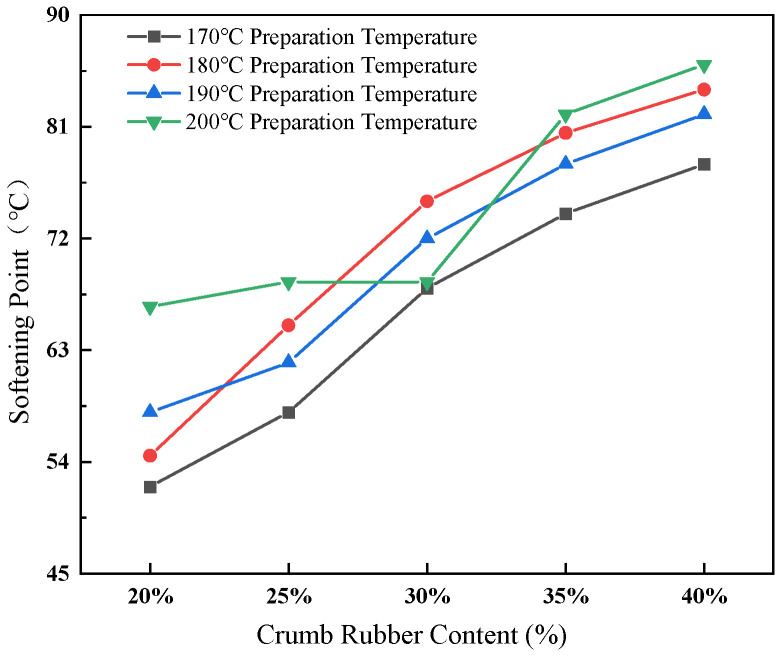
Effect of crumb rubber content and preparation temperature on softening point of rubberized asphalt.

The effect of crumb rubber content and preparation temperature on softening point is shown in Figure 4.

Figure 4 illustrates that the softening point exhibits a marked increase as both the preparation temperature and the rubber content rise. Specifically, at a preparation temperature of 180 °C, the softening point increased from 54.5 °C at 20% rubber content to 84.0 °C at 40% rubber content, indicating a significant enhancement in high-temperature stability. These findings suggest that the incorporation of rubber substantially improves the material’s resistance to permanent deformation. Li et al. [38] found that when the crumb rubber content ranged from 16% to 28%, the softening point increased with increasing rubber content, reaching approximately 70 °C at a rubber content of 28%, which is comparable to the softening point measured in this study at a rubber content of 30%.

**Figure 5:**
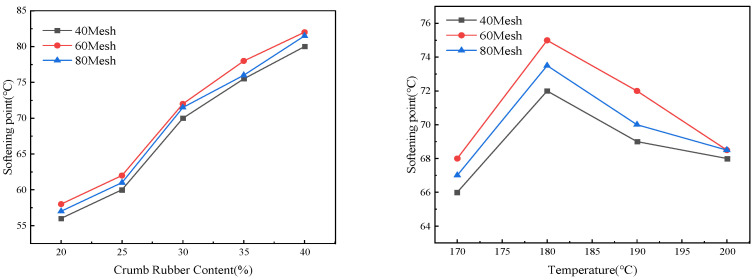
Effect of crumb rubber mesh size on softening point.

To further elucidate the influence mechanism of crumb rubber particle size distribution on high-temperature performance and to reveal its variation patterns under the combined effects of temperature and content, this study designed two sets of comparative experiments based on the previously optimized process. First, with the test temperature fixed at 190 °C, the effect of rubber powder mesh number size on the softening point was investigated at different rubber contents. Second, with the rubber content fixed at 30%, the mesh number at the size softening point under different conditions was examined. The experimental results are presented in Figure 5.

As shown in Figure 5, the mesh size of crumb rubber exhibits a consistent influence on the variation trend of the softening point. At 190 °C, the softening point continued to increase significantly with higher rubber content. Under a fixed 30% rubber content, the softening points of all particle sizes reached their peak at 180 °C and then declined at 200 °C, which is consistent with the previously observed phenomenon that excessive temperature leads to performance degradation. At the same content and temperature, the 60-mesh crumb rubber generally exhibited a higher softening point than other sizes, indicating that a moderate particle size is conducive to forming a more complete network structure. Therefore, 60-mesh crumb rubber was selected for experimental study in this work.

(4) Brookfield viscosity at 180 °C

**Figure 6:**
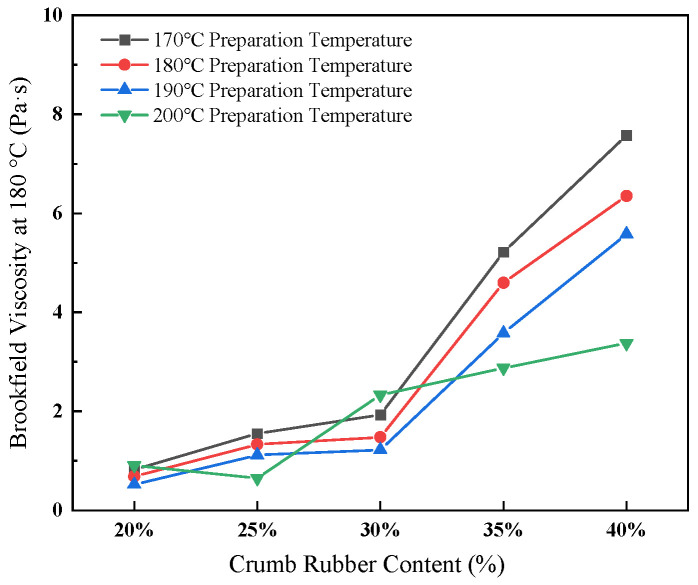
Effect of crumb rubber content and preparation temperature on viscosity at 180 °C.

The effect of crumb rubber content and preparation temperature on Brookfield viscosity at 180 °C is shown in Figure 6.

As illustrated in Figure 6, the viscosity exhibited a pronounced increase with rising rubber content, escalating from 0.820 Pa·s at 20% rubber concentration to 7.575 Pa·s at 40% rubber concentration at 170 °C. Conversely, an increase in the preparation temperature resulted in a reduction in viscosity; for instance, at 35% rubber content, viscosity decreased from 5.215 Pa·s at 170 °C to 2.874 Pa·s at 200 °C. These findings suggest that while higher rubber content enhances the viscoelastic properties, it adversely affects the material’s workability. In contrast, elevated temperatures improve fluidity but may compromise the structural stability of the composite.

(5) Sensitivity Analysis of the 30% Crumb Rubber Content

**Figure 7:**
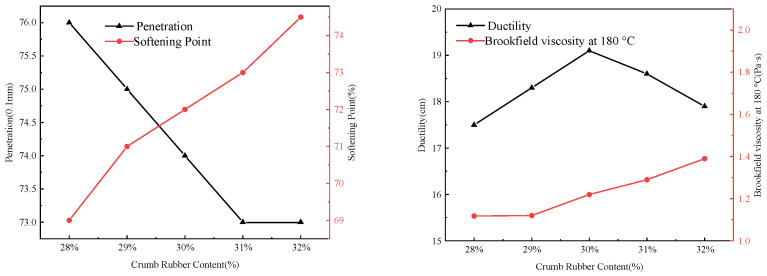
Test results of fundamental properties at varied crumb rubber content.

Basic performance tests were conducted at a preparation temperature of 190 °C with crumb rubber contents of 28%, 29%, 30%, 31%, and 32%. The results are presented in Figure 7.

As shown in Figure 7, within the dosage range of 28% to 32%, compared to the corresponding values at 30% dosage, the maximum variations in penetration, softening point, ductility, and Brookfield viscosity at 180 °C were 2.7%, 4.1%, 8.3%, and 13.9%, respectively. Within this narrow range, around 30%, all indicators exhibited relatively small fluctuations, suggesting that the performance of the binder remains relatively stable, with a favorable balance of properties observed at 30% crumb rubber content.

### 3.2. Aging Properties

(1) Penetration ratio after TFOT aging

**Figure 8:**
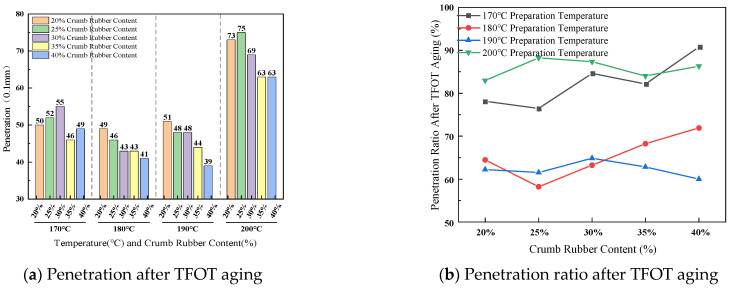
Effect of crumb rubber content and preparation temperature on penetration ratio after TFOT aging.

The effect of crumb rubber content and preparation temperature on penetration ratio after TFOT aging is shown in Figure 8.

As shown in Figure 8, the retained penetration ratio of rubberized asphalt containing 30% content remains at a level across preparation temperatures. This indicates that the three-dimensional network structure formed between the rubber particles and the asphalt at this content concentration is complete and stable, effectively inhibiting the volatilization of light components and oxidative cross-linking during aging, thereby significantly retarding performance. Slowing under the preparation temperature of 200 °C, although the retained penetration ratios of samples at various contents with various crumb rubber increased to a range of 83–88%, a genuine improvement was observed in aging resistance. This anomalous phenomenon is closely related to the pronounced thermal degradation of crumb rubber induced by high temperature: partially, the elevated rigid fragments and small-molecule polar substances generated during degradation act as filler-like components that physically harden the asphalt. Consequently, this leads to the disruption of the rubberized asphalt and the loss of interfacial weakening, increasing material brittleness. Thus, the penetration values remain relatively high even after aging.

(2) Ductility difference after TFOT aging

**Figure 9:**
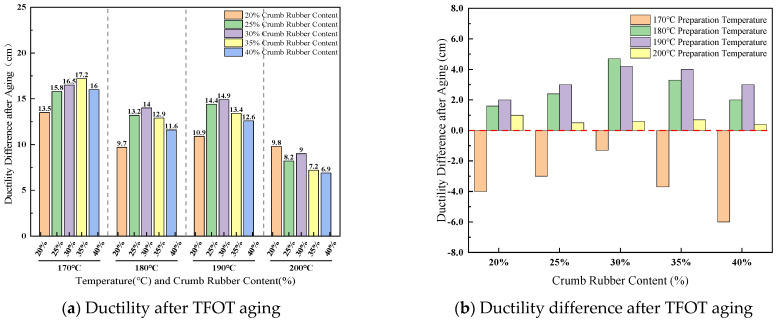
Effect of crumb rubber content and preparation temperature on ductility difference.

The effect of crumb rubber content and preparation temperature on ductility difference before and after TFOT aging is presented in Figure 9.

As shown in Figure 9, at a temperature of 170 °C, the ductility after aging is generally higher than that before, with the most pronounced improvement observed in the mid-to-high content of 30–40%. This is 30–40%. At this temperature, the rubber retains a certain capacity and structural reorganization during the aging process, exhibiting a characteristic of “secondary development.” When the temperature is between 180 °C and 190 °C, the ductility after aging is generally lower than that before aging. For example, at 190 °C and 30% content, it decreases from 19.1 cm to 30%. This suggests that within this temperature range, thermo-oxidative aging becomes dominant, the hardening effect of the thermo-oxidative matrix is significant, and the protective role of the rubber network gradually diminishes. Under the preparation condition of 200 °C, both the pre-aging and post-aging ductility values remain at °C, low levels, pre-aging and post-aging between them, and ductility is low as the content increases. This contrasts with the “abnormally high” retained penetration ratio observed at this temperature, revealing an intrinsic contradiction in the high-temperature aging mechanism: although severe degradation of the rubber can increase the penetration retention due to the formation of rigid fragments, it simultaneously leads to a sharp deterioration in material ductility, manifesting as an intensified macroscopic embrittlement trend.

(3) Softening point difference after TFOT aging

**Figure 10:**
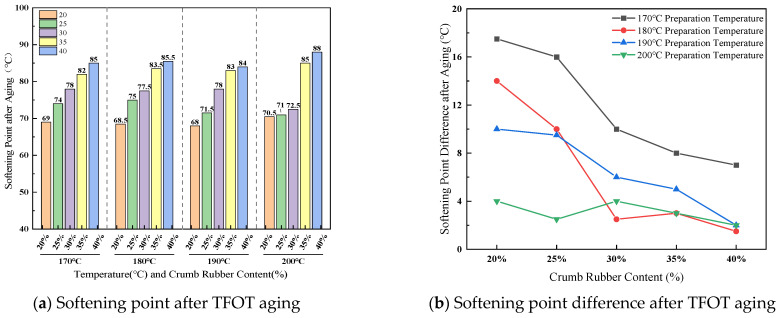
Effect of crumb rubber content and preparation temperature on softening point difference.

The effect of crumb rubber content and preparation temperature on softening point difference before and after TFOT aging is shown in Figure 10.

As shown in Figure 10, within the preparation temperature range of 170 °C to 190 °C, the softening point generally increases after aging, confirming short-term thermo-oxidative exposure. However, the extent of increase shows that this exhibits non-monotonicity with respect to temperature. For example, at 30% rubber content, from 68.0 °C to 78.0 °C at 170 °C, from 75.0 °C to 77.0 °C at 180 °C, and from 72.0 °C to 78.0 °C at 190 °C. This indicates that within this temperature window, the aging behavior is jointly governed by oxidation of the asphalt matrix range, and the state of the rubber is governed. Higher rubber content (≥30%) consistently maintains a higher absolute softening point value after aging, demonstrating that (≥30%) rubber results in the structural basis for preserving high-temperature stability. When the preparation temperature rises to 200 °C, the change in maintaining long-term high temperature and after aging is minimal, indicating that the high temperature has already caused severe thermal degradation of the rubber. The resulting rigid fragments elevated the material close to its hardening limit already during the preparation stage, leaving little potential for further aging.

(4) Brookfield viscosity at 180 °C ratio after TFOT aging

**Figure 11:**
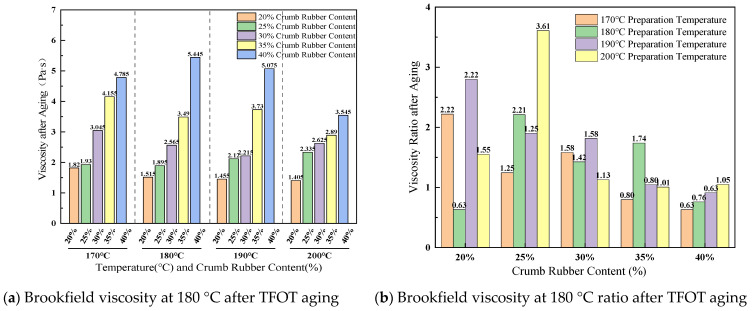
Effect of crumb rubber content and preparation temperature on viscosity ratio after TFOT aging.

The effect of crumb rubber content and preparation temperature on Brookfield viscosity at 180 °C ratio after TFOT aging is shown in Figure 11.

As illustrated in Figure 11, at low to moderate rubber concentrations (20–30%), aging resulted in a substantial increase in viscosity compared to the pre-aging state. For example, at 170 °C and 30% rubber content, the viscosity increased from 1.925 Pa·s to 3.045 Pa·s, representing a 58.2% rise. This finding suggests that within this concentration range, the rubber network remains effective in enhancing the internal resistance of the system during aging, thereby delaying the precipitation and flow of lighter components. Conversely, at 200 °C, the change in viscosity before and after aging was markedly attenuated. Specifically, at 40% rubber content, the viscosity exhibited only a slight increase of approximately 5%, from 3.375 Pa·s. This behavior contrasts distinctly with that observed at lower temperatures, indicating that elevated preparation temperatures induce significant thermal degradation of the rubber phase. As a result, the network structure is likely disrupted during the preparation process, substantially diminishing its capacity for further evolution during subsequent short-term aging.

In summary, aging tests revealed that binders synthesized at 190 °C exhibited the most optimal balance of properties, facilitating adequate rubber swelling while minimizing degradation.

It should be noted that the Thin Film Oven Test (TFOT) employed for short-term aging in this study involves a static pan configuration. Due to the high viscosity of rubberized binders, particularly at elevated crumb rubber contents, the static method may lead to uneven aging and potential skin formation compared to the rolling film method. However, as all samples were subjected to identical aging conditions, the comparative trends observed across different rubber contents and preparation temperatures remain reliable and provide meaningful insights into the aging behavior of the binders.

### 3.3. Rheological Properties

To further assess the high-temperature rutting resistance and viscoelastic properties, Dynamic Shear Rheometer (DSR) tests were performed at 190 °C with a rubber content of 30%. The findings from the temperature sweep tests are presented in Table 8.

(1) Complex shear modulus (|G*|)

**Figure 12:**
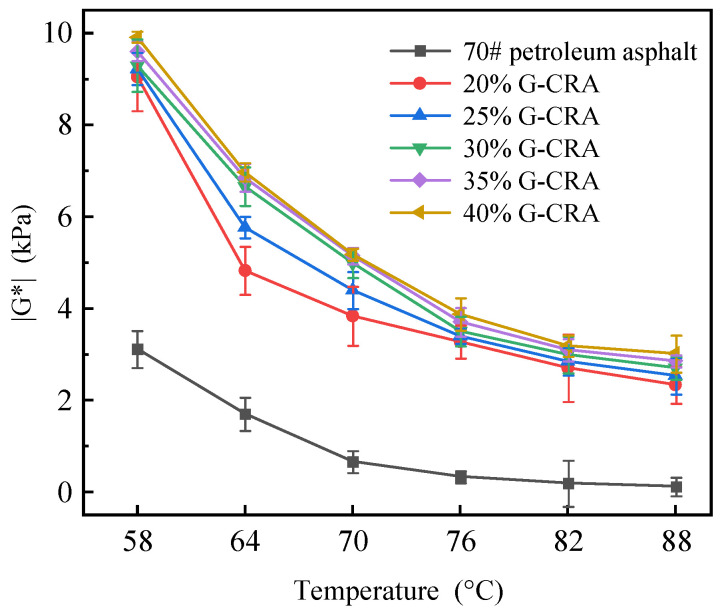
Complex shear modulus (|G*|) of base asphalt and the CRA at different temperatures.

The complex shear modulus (|G*|) of base asphalt and the CRA at different temperatures is shown in Figure 12.

As illustrated in Figure 12, the complex shear modulus (|G*|) of the base asphalt demonstrated a marked decline with increasing temperature, exhibiting a pronounced reduction beyond 76 °C, indicative of its thermal sensitivity. Conversely, the CRA samples displayed substantially higher |G*| values, which escalated in proportion to the rubber content. For example, at 70 °C, the base asphalt exhibited a |G*| of merely 0.67 kPa, whereas the CRA containing 30% rubber attained a |G*| of 4.40 kPa, and the 40% rubber CRA further increased to 5.18 kPa. These findings suggest that the incorporation of crumb rubber facilitates the formation of a spatial network within the binder matrix, thereby enhancing its elasticity and resistance to shear deformation.

The observed enhancement is attributed to the unique physical cross-linking provided by the crumb rubber. The swollen rubber phase acts as elastic nodes that interlock to form a percolated three-dimensional network throughout the asphalt matrix. This network contributes to the significant increase in |G*| through physical interactions. Importantly, the inherent high elasticity of the rubber polymer allows it to accommodate substantial deformation under shear loading, resulting in improved rheological shear resistance as measured by the Dynamic Shear Rheometer (DSR).

(2) Phase angle (δ)

**Figure 13:**
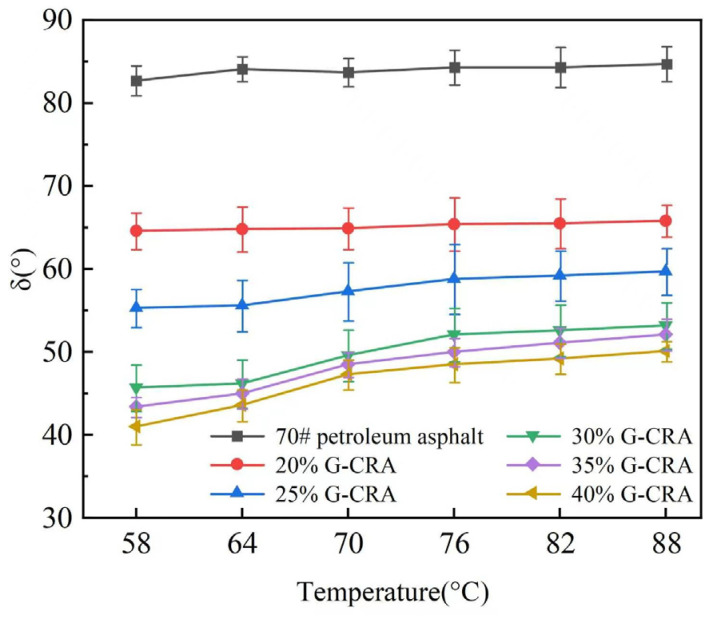
Phase angle (δ) of base asphalt and the CRA at different temperatures.

Phase angle (δ) of base asphalt and the CRA at different temperatures is shown in Figure 13.

Figure 13 illustrates that the δ values of the base asphalt ranged from 82 to 85 °C, signifying a predominantly viscous behavior. In contrast, the δ values of the CRA were significantly lower and exhibited a decreasing trend with increasing rubber content. For instance, at 76 °C, the δ value decreased from 84.3 °C for the base asphalt to 49.6 °C for the CRA containing 30% rubber and further declined to 47.3 °C for the CRA with 40% rubber. These reduced δ values suggest enhanced elasticity and improved recovery capacity under load, which contribute to greater resistance against permanent deformation.

(3) Rutting factor (|G*|/sinδ)

The rutting factor has been clarified as being evaluated on unaged binders, and the sentence has been revised accordingly.

|G*|/sin δ is a key rheological parameter used to evaluate the high-temperature resistance to permanent deformation of asphalt binders. As shown in Figure 14, the |G*|/sinδ of the asphalt is markedly significantly higher than that of the base asphalt and increases continuously with higher crumb rubber content. For instance, at 64 °C, the |G*|/sinδ of |G*|/sin δ base asphalt is only 1.72 kPa, whereas that of rubberized asphalt with 30% content reaches 30% crumb rubber, and the value for the 40% content is elevated to It increases noteworthy that even Notably, at 76–82 °C, the high-temperature asphalt maintains relatively high |G*|/sinδ values. This indicates, from a |G*|/sin δ perspective, that the incorporation of crumb rubber substantially enhances the binder’s potential to resist shear deformation under ambient temperatures. Comprehensive analysis reveals that, at the temperatures.

On a comprehensive scale, the addition of crumb rubber significantly improves the high-temperature rheological performance of asphalt through high-temperature formation of an elastic network. This is concretely manifested as enhanced stiffness; improvement is proportionally increased, and a higher improvement in the deformation resistance index marked enhancement should be observed (|G*|/sin δ). The rheological performance of the binder is a necessary prerequisite for the asphalt mixture to achieve satisfactory asphalt performance (e.g., rutting resistance). However, the ultimate road performance must be evaluated through a mixture design to validate pavement structure.

(4) Viscoelastic Analysis Using the Black Diagram

**Figure 15:**
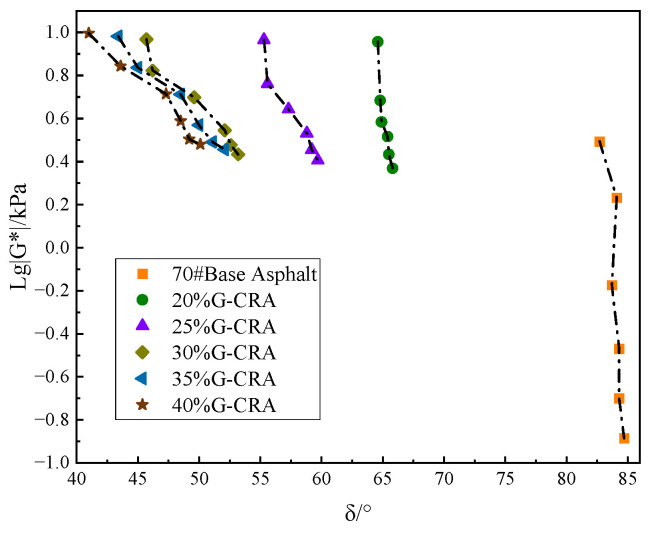
Black diagram illustrating the relationship between the phase angle (δ) and the logarithm of the complex modulus (lg |G*|).

The Black diagram, which illustrates the relationship between the phase angle (δ) and the logarithm of the complex modulus (lg |G*|), is shown in Figure 15.

As shown in Figure 15, the data points for the base asphalt are located in the lower right region of the diagram, exhibiting typical viscosity-dominant characteristics. Upon incorporation of deodorized crumb rubber, the curves of all modified asphalt binders systematically shift toward the upper left, indicating a synergistic enhancement in both the elastic component (reduced δ) and the resistance to deformation (increased |G*|). The trend displayed in the figure reveals a clear dependence on rubber content. At a rubber content of 30%, the curve exhibits a noticeable shift; further increasing the content to 40% results in highly overlapping curves with minimal additional displacement. This suggests that the viscoelastic improvement tends to stabilize beyond 30% rubber content. All data points for the modified asphalt form a smooth, continuous single curve, consistent with the behavior of a thermorheologically simple material, confirming that the rubber phase and the asphalt matrix have formed a homogeneous and stable new phase.

### 3.4. Microstructural Analysis and Mechanism Verification via Scanning Electron Microscopy (SEM)

**Figure 16:**
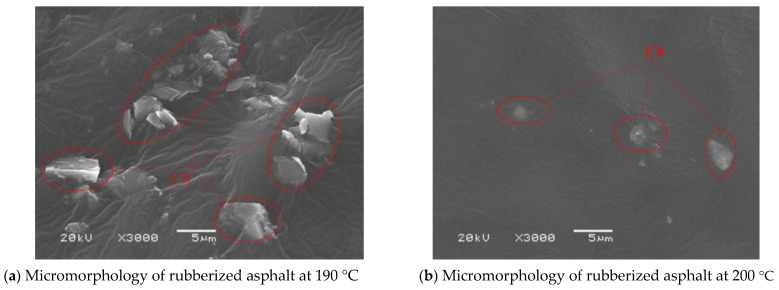
Micromorphology of rubberized asphalt under different temperatures.

To investigate the mechanisms underlying the effects of high temperature on the performance of rubberized asphalt at the microscopic scale, this section employs scanning electron microscopy (SEM) to analyze the morphology of samples under key processing conditions. Rubberized asphalt specimens containing 30% crumb rubber, prepared at 190 °C and 200 °C, respectively, were selected for comparative observation. The aim is to objectively reveal and elucidate the evolution of temperature-induced rubber’s internal structure and the changes in its interfacial characteristics with the asphalt matrix. The corresponding micromorphologies under these two conditions are shown in Figure 16.

As shown in Figure 16, in the sample prepared at 190 °C, the °C exhibits a well-integrated structure that is integrated into the surrounding asphalt matrix. This indicates that, at an appropriate crumb rubber, sufficient undergoes and forms a composite structure with the asphalt, dominated by primarily driven entanglement and adsorption. In sharp contrast, the sample prepared at 200 °C exhibits clear signs of microstructural deterioration, where microstructural rubber shows degradation and is released from the asphalt.

**Figure 17:**
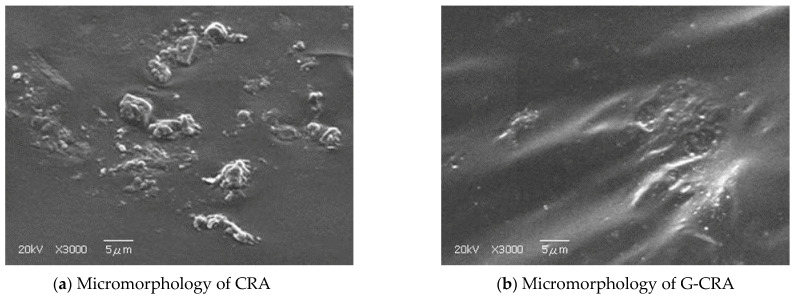
Micromorphology of CRA and G-CRA under the same preparation conditions (30% crumb rubber content, 190 °C).

To further elucidate the microstructural effect of the deodorization process, comparative SEM observations were conducted on conventional CRA and G-CRA prepared under the same optimal conditions (30% crumb rubber content, 190 °C preparation temperature). As shown in Figure 17a, the conventional CRA exhibits a relatively rough and heterogeneous morphology, with irregular rubber particles partially dispersed within the asphalt matrix. In contrast, Figure 17b reveals that the G-CRA presents a more uniform and compact microstructure, characterized by better interfacial bonding between the deodorized rubber particles and the surrounding asphalt. The surface of the deodorized rubber appears smoother and more intimately embedded in the binder, which may contribute to improved mechanical compatibility and enhanced fume adsorption capacity. These direct visual comparisons substantiate that the deodorization treatment not only modifies the surface characteristics of the rubber but also promotes more favorable interaction with the asphalt matrix under identical preparation conditions.

## 4. Effect of Crumb Rubber Content on Fume Emissions

The results of fume emission tests for conventional CRA and the G-CRA are summarized in Table 9. The results of the two-factor analysis of variance among the gases in Table 10.

As shown in Table 10, both crumb rubber type and content have a significant effect on the emissions of various gases (*p* < 0.01), indicating that crumb rubber type and content are key controlling factors influencing fume emissions.

(1) H_2_S emissions

**Figure 18:**
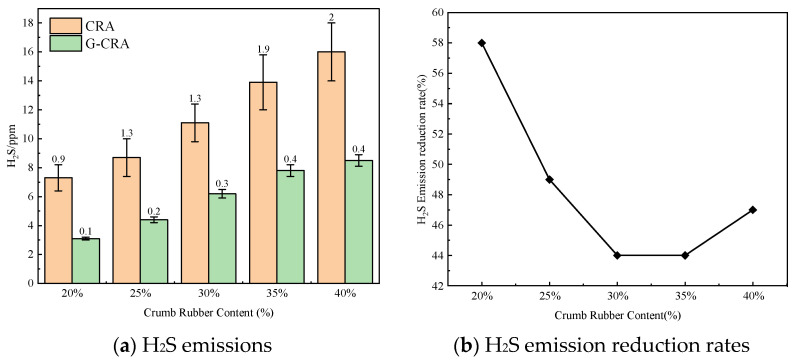
Effect of crumb rubber content on H_2_S emissions of the CRA and the G-CRA.

The effect of crumb rubber content on H_2_S emissions of the CRA and the G-CRA is shown in Figure 18.

As illustrated in Figure 18, hydrogen sulfide (H_2_S) emissions from the CRA exhibited a significant increase in relation to the rubber content, rising from 7.3 ppm at 20% rubber content to 16.0 ppm at 40%. In contrast, the emissions from G-CRA varied between 3.1 and 8.5 ppm, reflecting reduction rates of 44% to 58%. To contextualize these results, a comparison with existing literature is provided. Liu et al. [39] investigated the use of an organic-inorganic composite inhibitor in rubber-modified asphalt and reported that the addition of 0.25% organic inhibitor and 2.5% diatomite reduced the H_2_S concentration from 7 ppm in conventional rubberized asphalt to 3 ppm, achieving a reduction rate of 57%. Despite differences in material composition and test conditions, the reduction rate of 57% reported by Liu et al. is highly consistent with the maximum reduction of 58% achieved by G-CRA in this study. These findings suggest that the use of deodorized rubber substantially mitigates sulfide emissions, thereby diminishing the intensity of unpleasant odors during construction activities.

(2) VOC emissions

**Figure 19:**
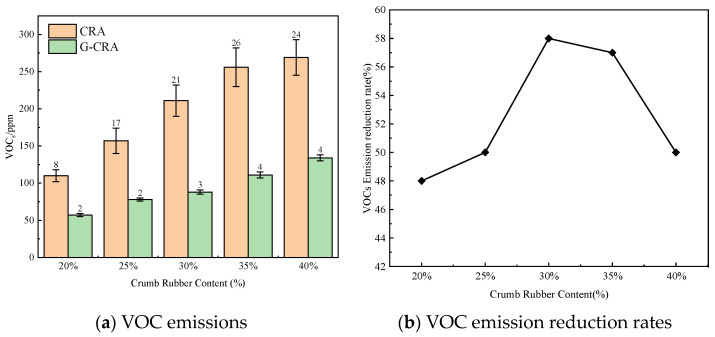
Effect of crumb rubber content on VOC emissions of the CRA and the G-CRA.

The effect of crumb rubber content on VOC emissions of the CRA and the G-CRA is shown in Figure 19.

As shown in Figure 19, VOC emissions from conventional CRA increased with rising rubber content, from 110 ppm to 269 ppm. In contrast, emissions from G-CRA ranged from 57 ppm to 134 ppm, corresponding to an overall reduction rate of 48–58%. For comparison, Zhang et al. [20] reported that coating crumb rubber with waste cooking oil (WCO) reduced the average VOC concentration by 20.8%. These findings indicate that the deodorization treatment effectively reduces the volatilization of light components in asphalt, thereby lowering the emission of organic odor pollutants.

(3) NO emissions

**Figure 20:**
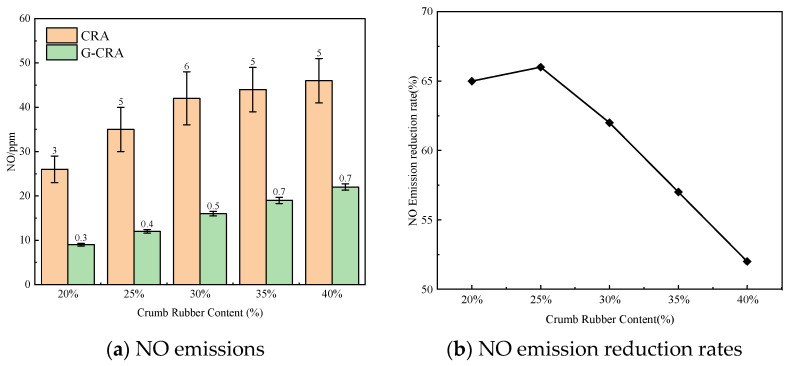
Effect of crumb rubber content on NO emissions of the CRA and the G-CRA.

The effect of rubber content on NO emissions of the CRA and G-CRA is shown in Figure 20.

As illustrated in Figure 20, the nitrogen oxide (NO) emissions from the CRA varied between 26 and 46 ppm, exhibiting a modest increase corresponding to the rubber content. In contrast, the G-CRA samples displayed significantly lower NO emission levels, ranging from 9 to 22 ppm, which corresponds to a reduction rate of 52 to 66%. These findings indicate that the use of deodorized rubber substantially inhibits the formation of nitrogen oxides.

(4) CO emissions

**Figure 21:**
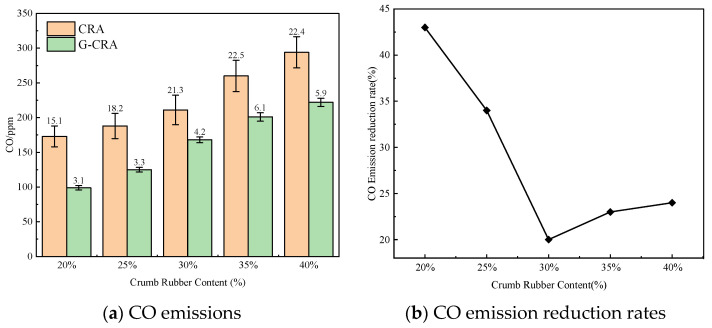
Effect of crumb rubber content on CO emissions of the CRA and the G-CRA.

The effect of rubber content on CO emissions of the CRA and G-CRA is shown in Figure 21.

Figure 21 demonstrates that carbon monoxide (CO) emissions from the CRA increased from 173 ppm to 294 ppm as the rubber content increased. In contrast, the CO emissions for the G-CRA varied between 99 ppm and 222 ppm. The observed reduction rates ranged from 20% to 43%, suggesting that the deodorization process moderately mitigates the production of incomplete combustion byproducts.

(5) SO_2_ emissions

**Figure 22:**
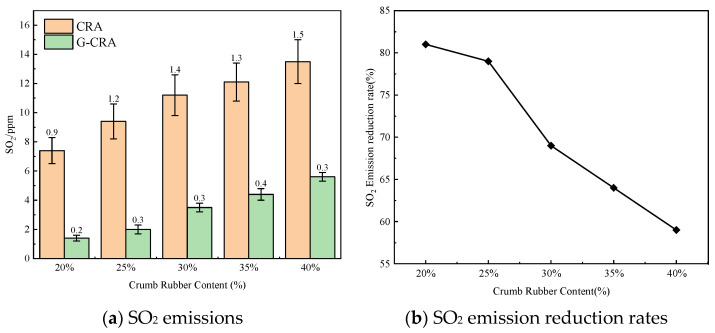
Effect of crumb rubber content on SO_2_ emissions of the CRA and the G-CRA.

The effect of rubber content on SO_2_ emissions of the CRA and G-CRA is shown in Figure 22.

As illustrated in Figure 22, sulfur dioxide (SO_2_) emissions from the CRA increased from 7.4 ppm to 13.5 ppm with higher content levels. In contrast, the G-CRA exhibited significantly lower emissions, ranging from 1.4 to 5.6 ppm, corresponding to reduction rates between 59% and 81%. These findings underscore the enhanced efficacy of deodorized rubber in mitigating sulfur-based pollutant emissions.

The superior inhibition of SO_2_ over CO by deodorized crumb rubber is mainly attributed to their fundamental differences in physical adsorption and chemical capture. Physically, SO_2_, as a polar molecule with a relatively high boiling point, benefits from the increased specific surface area and porous structure of the deodorized rubber, which enhances dipole interactions and pore condensation adsorption. In contrast, nonpolar CO is only weakly adsorbed via dispersion forces. Chemically, the deodorization process exposes numerous active sites on the rubber surface, where SO_2_ can undergo specific reactions such as acid–base neutralization and addition, forming stable compounds. CO, being chemically inert, hardly participates in similar reactions. Thus, deodorized crumb rubber achieves efficient inhibition of SO_2_ through the synergistic interaction of “polarity-enhanced physical adsorption” and “SO_2_-specific chemical conversion”, while exhibiting significantly weaker capture of CO.

(6) CO_2_ emissions

**Figure 23:**
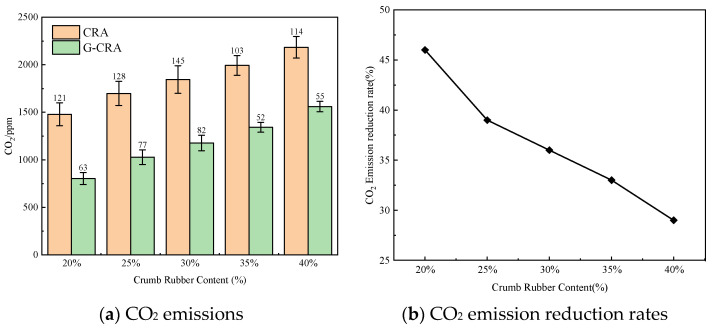
Effect of crumb rubber content on CO_2_ emissions of the CRA and the G-CRA.

The effect of rubber content on CO_2_ emissions of the CRA and G-CRA is shown in Figure 23.

Figure 23 illustrates that the carbon dioxide (CO_2_) emissions associated with the CRA ranged from 1479 to 2184 ppm, whereas the G-CRA exhibited reduced emissions between 803 and 1560 ppm, corresponding to a reduction rate of 29 to 46%. These findings suggest that the use of deodorized rubber not only mitigates harmful pollutants but also effectively decreases greenhouse gas emissions.

(7) Particulate matter (PM) emissions

**Figure 24:**
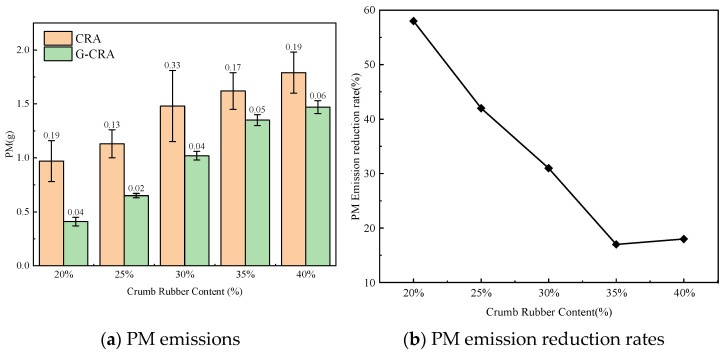
Effect of crumb rubber content on particulate matter emissions of the CRA and the G-CRA.

The effect of rubber content on PM emissions of the CRA and G-CRA is shown in Figure 24.

As illustrated in Figure 23, particulate matter (PM) emissions from the CRA increased proportionally with the rubber content. In contrast, the G-CRA exhibited significantly lower PM emissions across all rubber content levels. The observed reduction rates, ranging between 30% and 50%, indicate that the deodorization process effectively mitigates the release of PM_10_ and PM_2.5_, thereby contributing to enhanced air quality at construction sites.

Based on the fume emission test results described above, it is hypothesized that the deodorization process achieves efficient emission reduction through a synergistic mechanism combining physical adsorption and chemical reactions. At the physical level, the significant reduction in fume emissions observed in this study corresponds with the adsorption properties of the modified components within the asphalt. Previous research indicates that porous materials, such as organically modified montmorillonite and biochar, can effectively adsorb volatile organic compounds (VOCs) from asphalt fumes via interlayer pores or surface physical structures, thereby achieving substantial emission reduction [40]. At the chemical level, specific active components in the deodorant may further react with odorous sulfur- and nitrogen-containing compounds released during the thermal decomposition of asphalt, converting them into non-volatile or low-odor stable products [41]. This combination of chemical transformation and physical adsorption is expected to collectively explain the exceptionally broad-spectrum emission reduction performance of the deodorized crumb rubber.

## 5. Conclusions

This study systematically investigated the effects of crumb rubber content (20–40%) and preparation temperature (170–200 °C) on the conventional properties, Brookfield viscosity at 180 °C, short-term aging behavior (TFOT), rheological characteristics, and fume emissions of high-content deodorized rubberized asphalt (G-CRA). The main conclusions are as follows:

(1) Conventional properties of G-CRA were significantly influenced by both rubber content and preparation temperature. Penetration increased with rising temperature but decreased with increasing rubber content. Ductility showed a non-linear response, peaking at 19.1 cm with 30% rubber content at 190 °C. The softening point increased with both rubber content and temperature, indicating improved high-temperature stability. Brookfield viscosity at 180 °C increased markedly with rubber content but decreased at higher preparation temperatures. Short-term aging results revealed distinct behaviors: binders prepared at 170 °C exhibited high penetration retention and negative ductility differences due to secondary swelling, whereas those prepared at 180–190 °C showed greater aging sensitivity; at 200 °C, evident rubber degradation led to nearly complete loss of ductility.
(2) Rheological analysis showed that both the complex shear modulus (|G|) and rutting factor (|G|/sinδ) increased substantially with rubber content, while phase angle (δ) decreased, reflecting enhanced elasticity and rutting resistance. Optimal rheological performance was observed at rubber contents between 30% and 35%, significantly outperforming the base asphalt.
(3) G-CRA demonstrated effective emission reduction. Compared with conventional CRA, G-CRA reduced emissions of hydrogen sulfide (H_2_S), volatile organic compounds (VOCs), nitrogen oxides (NO), and sulfur dioxide (SO_2_) by more than 40%, with SO_2_ reduction reaching up to 81%. A favorable balance between performance and emission mitigation was achieved at rubber contents of 30–35%.
(4) Based on a comprehensive evaluation of conventional properties, Brookfield viscosity, short-term aging behavior, rheological characteristics, and emission reductions, the optimal preparation conditions were identified as 30% rubber content and a preparation temperature of 190 °C, with 1 h of shearing followed by 2 h of swelling. The use of deodorized crumb rubber enables synergistic improvement in both performance and environmental benefits, providing theoretical and practical support for sustainable pavement construction.

In future works, it is recommended to systematically perform long-term aging and fatigue assessments of rubberized asphalt, integrating chemical and structural characterization methodologies such as Fourier transform infrared spectroscopy, thermogravimetric analysis, and scanning electron microscopy. Additionally, analytical techniques, including gas chromatography–mass spectrometry, should be employed to comprehensively elucidate the fundamental mechanisms through which deodorized crumb rubber mitigates fume emissions.

## Figures and Tables

**Figure 14:**
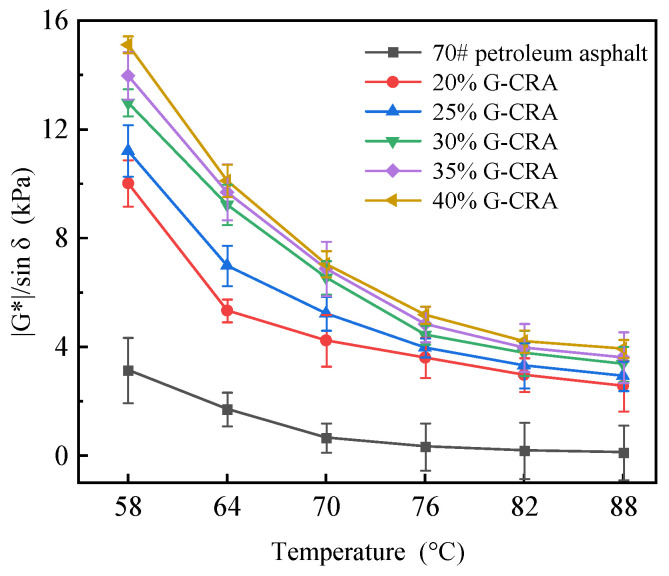
Rutting factor (|G*|/sin δ) of base asphalt and the CRA at different temperatures.

**Table 1:** Main technical properties of base asphalt.

Test	Specification	Result	Test Method
Penetration (25 °C, 5 s, 100 g) (0.1 mm)	60~80	66	T 0604-2011
Softening point (°C)	≥45	47	T 0606-2011
Ductility (15 °C, 1 cm·min^−1^) (cm)	≥100	>100	T 0605-2011
Ductility (5 °C, 1 cm·min^−1^) (cm)	-	0	T 0605-2011
Brookfield viscosity at 135 °C (Pa·s)	≥0.16	0.26	T 0625-2011

**Table 2:** Main technical properties of conventional crumb rubber.

Test Item	Result
Ash content (%)	7
Iron content (%)	0.003
Fiber content (%)	0.2
Rubber hydrocarbon content (%)	55
Acetone extract (%)	12
Carbon black content (%)	30

**Table 3:** Main technical properties of G-CR.

Test Item	Result
Ash content (%)	8
Iron content (%)	0
Fiber content (%)	0.1
Rubber hydrocarbon content (%)	49
Acetone extract (%)	14
Carbon black content (%)	32

**Table 4:** Technical properties of aromatic oil additive.

Test Item	Result
Flash point (°C)	265
Kinematic viscosity (%)	30
Aromatic content (%)	80
Sulfur content (%)	0.5
Volatile matter (%)	0
Moisture (%)	0
Ash content (%)	0.01
Density (g/cm^3^)	0.99
Color	Dark green

**Table 5:** Preparation parameters of rubberized asphalt.

Stage	Temperature (°C)	Time (h)	Stirring Speed (r/min)
Premixing	Test temperature	0.5	600
Shearing	Test temperature	1	5000
Swelling	180	2	600

**Table 6:** Influence of crumb rubber content and preparation temperature on the properties.

Preparation Temperature (°C)	Rubber Content (%)	Rubberized Asphalt Properties				After TFOT			
		Penetration (0.1 mm)	Ductility (cm)	Softening Point (°C)	Viscosity (Pa·s)	Penetration (0.1 mm)	Ductility (cm)	Softening Point (°C)	Viscosity (Pa·s)
170	20	64	9.5	52.0	0.820	50	13.5	69.0	1.820
	25	68	12.8	58.0	1.550	52	15.8	74.0	1.930
	30	65	15.2	68.0	1.925	55	16.5	78.0	3.045
	35	56	13.5	74.0	5.215	46	17.2	82.0	4.155
	40	54	10	78.0	7.575	49	16.0	85.0	4.785
180	20	76	11.3	54.5	0.685	49	9.7	68.5	1.515
	25	79	15.6	65.0	1.330	46	13.2	75.0	1.895
	30	68	18.7	75.0	1.475	43	14.0	77.0	2.565
	35	63	16.2	80.5	4.595	43	12.9	83.5	3.490
	40	57	13.6	84	6.350	41	11.6	85.5	5.445
190	20	82	12.9	58.0	0.520	51	10.9	68.0	1.455
	25	78	17.4	62.0	1.115	48	14.4	72.0	2.120
	30	74	19.1	72.0	1.220	48	14.9	78.0	2.215
	35	70	17.4	78.0	3.575	44	13.4	83.0	3.730
	40	65	15.6	82.0	5.575	39	12.6	84.0	5.075
200	20	88	10.8	66.5	0.906	73	9.8	67.0	1.405
	25	85	8.7	68.5	0.647	75	8.2	70.5	2.335
	30	79	9.6	68.5	2.328	69	9.0	72.5	2.625
	35	75	7.9	82.0	2.874	63	7.2	85.0	2.890
	40	73	7.3	86.0	3.375	63	6.9	88.0	3.545

**Table 7:** Influence of crumb rubber concentration and processing temperature on the TFOT-aged characteristics.

Reparation Temperature (°C)	Rubber Content (%)	Retained Penetration Ratio (%)	Ductility Difference (cm)	Softening Point Difference (°C)	Viscosity Ratio
170	20	78	−4.0	17.5	2.2
	25	76	−3.0	16	1.2
	30	85	−1.3	10	1.6
	35	82	−3.7	8	0.8
	40	91	−6.0	7	0.6
180	20	64	1.6	14	2.2
	25	58	2.4	10	1.4
	30	63	4.7	2.5	1.7
	35	68	3.3	3	0.8
	40	72	2.0	1.5	0.9
190	20	62	2.0	10	2.8
	25	62	3.0	9.5	1.9
	30	65	4.2	6	1.8
	35	63	4.0	5	1.0
	40	60	3.0	2	0.9
200	20	83	1.0	4	1.6
	25	88	0.5	2.5	3.6
	30	87	0.6	4	1.1
	35	84	0.7	3	1.0
	40	86	0.4	2	1.1

**Table 8:** Temperature sweep results of base asphalt and deodorized crumb rubber asphalt (G-CRA).

Test Item	Test Temperature	70# Base Asphalt	The G-CRA				
			20%	25%	30%	35%	40%
δ (°C)	58	82.7	64.6	55.3	45.7	43.4	41.0
	64	84.1	64.8	55.6	46.2	45.0	43.6
	70	83.7	64.9	57.3	49.6	48.5	47.3
	76	84.3	65.4	58.8	52.1	50.0	48.5
	82	84.3	65.5	59.2	52.6	51.1	49.2
	88	84.7	65.8	59.7	53.2	52.1	50.1
|G*|(kPa)	58	3.12	9.05	9.22	9.29	9.60	9.91
	64	1.71	4.83	5.77	6.66	6.85	6.97
	70	0.67	3.84	4.40	4.99	5.15	5.18
	76	0.34	3.28	3.40	3.51	3.71	3.88
	82	0.20	2.71	2.85	3.00	3.10	3.19
	88	0.13	2.34	2.54	2.71	2.86	3.02
|G*|/sin δ (kPa)	58	3.15	10.02	11.21	12.98	13.97	15.11
	64	1.72	5.34	6.99	9.23	9.69	10.11
	70	0.67	4.24	5.23	6.55	6.88	7.05
	76	0.34	3.61	3.98	4.45	4.84	5.18
	82	0.20	2.98	3.32	3.78	3.98	4.21
	88	0.13	2.57	2.94	3.38	3.62	3.94

**Table 9:** Fume emission results of the conventional CRA and the G-CRA at different rubber contents.

Sample	H_2_S (ppm)	VOCs (ppm)	NO (ppm)	CO (ppm)	SO_2_ (ppm)	CO_2_ (ppm)	PM (g)
20%the CRA	7.3	110	26	173	7.4	1479	0.97
25%the CRA	8.7	157	35	188	9.4	1698	1.13
30%the CRA	11.1	211	42	211	11.2	1845	1.48
35%the CRA	13.9	256	44	260	12.1	1993	1.62
40%the CRA	16	269	46	294	13.5	2184	1.79
20%the G-CRA	3.1	57	9	99	1.4	803	0.41
25%the G-CRA	4.4	78	12	125	2	1028	0.65
30%the G-CRA	6.2	88	16	168	3.5	1177	1.02
35%the G-CRA	7.8	111	19	201	4.4	1342	1.35
40%the G-CRA	8.5	134	22	222	5.6	1560	1.47

**Table 10:** The results of the two-factor analysis of variance among the gases.

Gas Type	Source of Difference	SS	df	MS	F	*p*-Value
H_2_S	Crumb Rubber Type	218.7	1	218.7	181.3	<0.01
	Crumb Rubber Content	204.6	4	51.2	42.4	<0.01
	Interaction	11.7	4	2.9	2.4	0.082
VOCs	Crumb Rubber Type	85,867.5	1	85,867.5	409.9	<0.01
	Crumb Rubber Content	55,267.2	4	13,816.8	65.9	<0.01
	Interaction	9276	4	2319.0	11.1	<0.01
NO	Crumb Rubber Type	3967.5	1	3967.5	326.6	<0.01
	Crumb Rubber Content	1054.2	4	263.6	21.7	<0.01
	Interaction	75	4	18.8	1.5	0.228
CO	Crumb Rubber Type	29,016.3	1	29,016.3	140.6	<0.01
	Crumb Rubber Content	61,312.2	4	15,328.1	74.3	<0.01
	Interaction	922.2	4	230.6	1.1	0.376
SO_2_	Crumb Rubber Type	404.067	1	404.1	468.8	<0.01
	Crumb Rubber Content	99.75	4	24.9	28.9	<0.01
	Interaction	3.558	4	0.9	1.0	0.415
CO_2_	Crumb Rubber Type	3,245,256	1	3,245,256.0	331.1	<0.01
	Crumb Rubber Content	1,881,703	4	470,425.8	48.0	<0.01
	Interaction	2659.2	4	664.8	0.1	0.991
PM	Crumb Rubber Type	1.306253	1	1.3	55.3	<0.01
	Crumb Rubber Content	3.735353	4	0.9	39.5	<0.01
	Interaction	0.084513	4	0.0	0.9	0.486

## Data Availability

The original contributions presented in this study are included in the article. Further inquiries can be directed to the corresponding authors.
